# The first high-quality chromosome-level genome of *Eretmochelys imbricata* using HiFi and Hi-C data

**DOI:** 10.1038/s41597-023-02522-3

**Published:** 2023-09-09

**Authors:** Yusong Guo, Jiao Tang, Zixuan Zhuo, Jingru Huang, Zhenli Fu, Jiahao Song, Min Liu, Zhongdian Dong, Zhongduo Wang

**Affiliations:** 1 Key Laboratory of Aquaculture in South China Sea for Aquatic Economic Animal of Guangdong Higher Education Institutes, Fisheries College, Guangdong Ocean University, Zhanjiang, 524088 China; 2 State Key Laboratory of Marine Environmental Science and College of Ocean & Earth Sciences, Xiamen University, Xiamen, Fujian China; 3 Guangdong Provincial Key Laboratory of Aquatic Animal Disease Control and Healthy culture, Fisheries College, Guangdong Ocean University, Zhanjiang, 524088 China

**Keywords:** DNA sequencing, Evolutionary genetics, Molecular biology

## Abstract

*Eretmochelys imbricata*, a critically endangered sea turtle inhabiting tropical oceans and protected across the world, had an unknown genome sequence until now. In this study, we used HiFi reads and Hi-C technology to assemble a high-quality, chromosome-level genome of *E. imbricata*. The genome size was 2,138.26 Mb, with contig N50 length of 123.49 Mb and scaffold N50 of 137.21 Mb. Approximately 97.52% of the genome sequence was anchored to 28 chromosomes. A total of 20,206 protein-coding genes were predicted. We also analyzed the evolutionary relationships, gene family expansions, and positive selection of *E. imbricata*. Our results revealed that *E. imbricata* diverged from *Chelonia mydas* 38 million years ago and had enriched olfactory receptors and aging-related genes. Our genome will be useful for studying *E. imbricata* and its conservation.

## Background & Summary

Sea turtles are a group with a long evolutionary history, having diverged for more than 100 million years^1^. Of the approximately 356 species of turtles worldwide^2^, only seven are sea turtles: the hawksbill turtle - *Eretmochelys imbruaria*, the green turtle - *Chelonia mydas*, the loggerhead turtle - *Caretta Caretta*, the olive ridley - *Lepidochelys olivacea*, the Kemp’s turtle - *Lepidochelys kempii*, the flatback turtle - *Natator depressius*, and the leatherback turtle - *Dermochelys coriacea*^3^. Sea turtles are widely distributed in global oceans and have highly migratory behavior, with migratory routes often spanning multiple seas and even oceans^4^. They are known for their remarkable survivability, reproductive capacity, and physiological diversity^5^. However, global sea turtle populations have been depleted in recent decades due to harvest for meat and eggs, commercial trade, fishery by-catch^6^, ecological degradation, and population gender disorders caused by global warming^7,8^. As a result, efforts to monitor, track, and protect sea turtles have increased in recent years.

*Eretmochelys imbricata*, commonly known as hawksbill turtle, is predominantly found in tropical and subtropical waters across the globe, and currently faces a very serious situation^9^. Among all globally distributed sea turtle species, it is the most endangered, and the IUCN has classified it as critically endangered (IUNC 2022). Despite concerted conservation efforts and interventions initiated since 1970, aimed at protecting and recovering *E. imbricata* populations, the species continues to face persistently low population levels^10,11^. Moreover, their significance in coral reef ecosystems cannot be understated, but the present global coral reef ecosystem faces severe degradation, further intensifying the threat to the survival of *E. imbricata*^12,13^. Conservation efforts for *E. imbricata* are particularly challenging due to their complex spatial structure and highly migratory nature^14,15^. The species needs may take decades to reach sexual maturity. Once mature, they return to their birthplace every few years to lay eggs, making it both difficult and costly to monitor their movements in the wild^16^. Most existing studies on *E. imbricata* primarily focus on counting nesting sites to assess their distribution^17^ and employing mitochondrial DNA haplotypes and microsatellite markers to examine their genetic structure^18,19^. Additionally, the development of Single Nucleotide Polymorphisms (SNPs) in *E. imbricata* has proved essential for evaluating their population structure^20,21^. However, despite these efforts, to date, there has been no reported genome assembly for *E. imbricata*.

In this study, we present the first high-quality, chromosome-level genome assembly of *E. imbricata*, achieved through PacBio HiFi and Hi-C sequencing technologies. The assembly resulted in a 2,138.26 Mb genome, with a contig N50 length of 123.49 Mb and a scaffold N50 of 137.21 Mb. Using Hi-C data, 97.52% of the assembled bases were successfully anchored to 28 chromosomes. This high-quality reference genome lays a robust groundwork for future population and conservation genetic studies of *E. imbricata*.

## Methods

### Sample collection and DNA extraction

An individual *E. imbricata* was obtained from the sea turtle rescue base on Naozhou Island, Zhanjiang City, Guangdong Province, China. A 10 mL blood sample was drawn from its jugular sinus and rapidly frozen for further analysis. Genomic DNA was extracted from the processed blood samples using the DNeasy Blood & Tissue Kit (Qiagen). The quality and quantity of the extracted DNA were assessed using a NanoDrop 2000 spectrophotometer (NanoDrop Technologies, Wilmington, DE, USA), a Qubit dsDNA HS assay kit on a Qubit 3.0 fluorometer (Life Technologies, Carlsbad, CA, USA), and 0.8% agarose gels.

### Library construction and sequencing

The DNA extracted from the blood was used for sequencing library construction using the PacBio SEQUEL Platform. For 20 kb template library preparation, ten micrograms (μg) of *E. imbricata* genomic DNA were utilized, following the manufacturer’s protocol with the BluePippin Size Selection system (Sage Science, Beverly, MA, USA). The PacBio single molecule real-time (SMRT) library was prepared using the SMRT bell express template prep Kit 2.0 (Pacific Biosciences, Menlo Park, CA, USA) and sequenced on the PacBio Sequel II platform in CCS mode. The raw data was converted into high-precision HiFi reads using the CCS workflow13 (v. 6.3.0, https://github.com/pacificbiosciences/unanimity) (parameters: - minPasses 3). A total of 30.11 Gb of HiFi reads with 27.26x coverage was generated, and the N50 value was 14,598 bp (Table 1).

**Table 1:** HiFi sequencing data statistics.

Data type	Sample	Total bases (Gbp)	Total number	Minimum length	Average length	Maximum length	N50
Polymerase read	B2_4	493.39	5,032,161	50	98,047	554,953	217,900
Subread	B2_4	492.06	35,056,699	50	14,037	554,953	14,715
HiFi read	B2_4	30.11	2,021,339	59	14,896	41,404	14,598

For Hi-C library preparation, the previously reported method^22^ was followed. Blood tissue was fixed with 2% formaldehyde, and the cross-linked DNA was digested with MboI enzyme. Biotin-labeled adapters were attached to the sticky ends of fragmented DNA. After reverse crosslinks by proteinase K (Thermo, Shanghai, China), DNA purification was performed using the QIAamp DNA Mini Kit (Qiagen) following the manufacturer’s instructions. The purified DNA was then sheared to a length of 300–500 bp to construct Hi-C libraries. A total of 186.13 G raw reads, which obtained from the MGI-SEQ. 2000 sequencing platform in paired-end 150 bp mode, were trimmed for sequencing adaptors and low-quality fragments using Trimmomatic (v0.39, parameters: LEADING:3 TRAILING:3 SLIDINGWINDOW:4:15 MINLEN:15). Finally, 181.16 Gb of high-quality Hi-C data were used to construct the chromosome-level genome. (Table 2).

**Table 2:** Hi-C sequencing data statistics.

Sample Name	Raw reads number	Raw bases (G)	Clean reads number	Clean bases(G)	Clean rate (%)	Q20(%)	Q30(%)	GC (%)
B2_11	620,423,025	186.13	608,641,938	181.16	98.1	97.74	91.92	45.46

For transcriptome sequencing, RNA was extracted from blood tissues using TRIzol reagent (Invitrogen, Waltham, MA, USA) following the manufacturer’s instructions. mRNA was then purified from the total RNA using poly-T oligo-attached magnetic beads. Sequencing libraries were generated from the purified mRNA using the V AHTS Universal V6 RNA-seq Library Kit for MGI (V azyme, Nanjing, China) with unique index codes following the manufacturer’s recommendations. The library quantification and size were assessed using Qubit 3.0 Fluorometer (Life Technologies, Carlsbad, CA, USA) and Bioanalyzer 2100 system (Agilent Technologies, CA, USA). Subsequently, sequencing was performed on the MGI-SEQ 2000 platform by Frasergen Bioinformatics Co., Ltd. (Wuhan, China).

### Genome survey and assembly

**Fig. 1:**
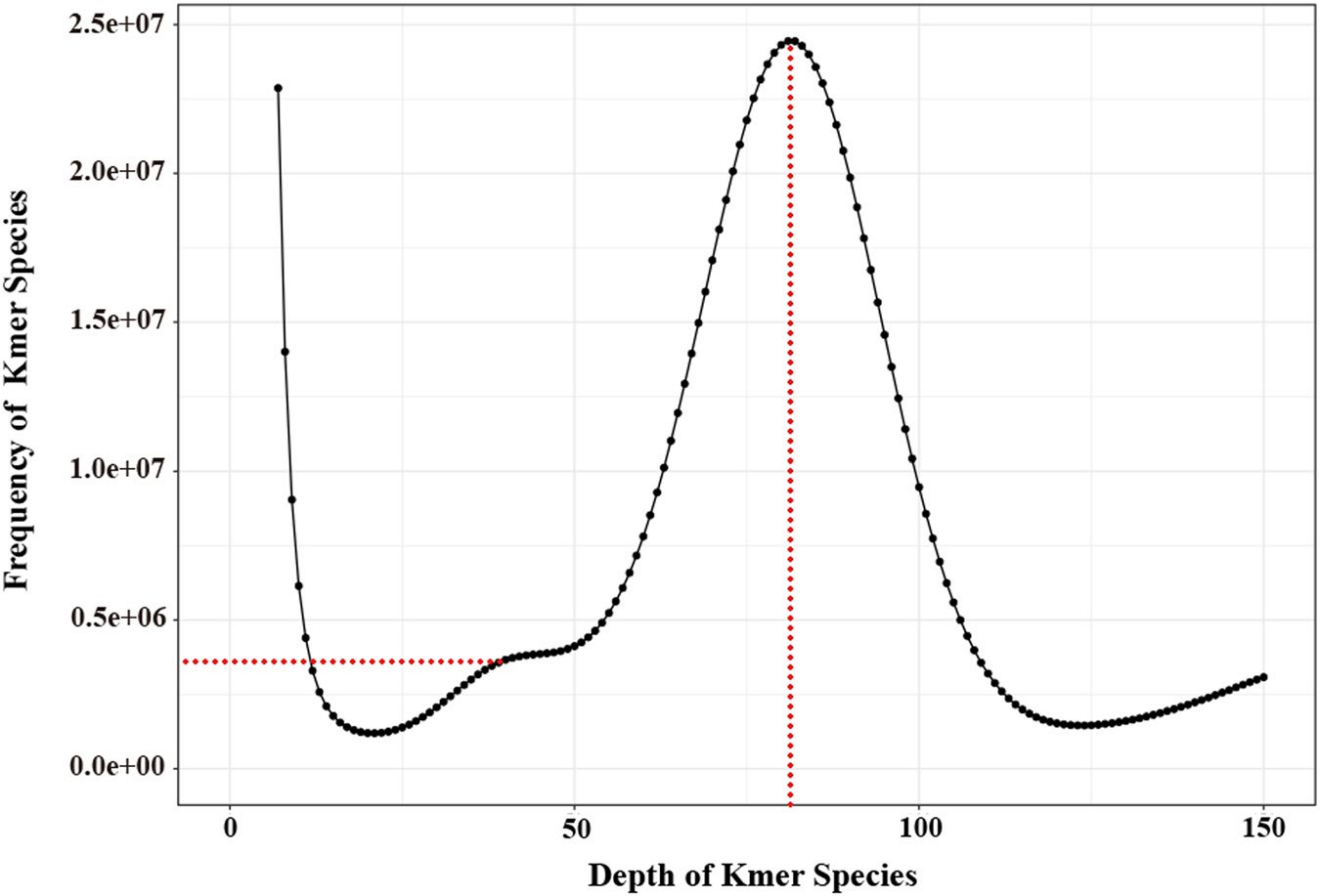
K-mer distribution of *E. imbricata*. Horizontal dotted line indicates heterozygosity rate, vertical dotted line represents a k-mer depth.

To estimate the genome size, heterozygosity, and repeat rate of *E. imbricata*, we employed the k-mer frequency method. The raw reads obtained from the DNBSEQ-T7 platform were quality-filtered using SOAPnuke (v2.1.0)^23^ (main parameters: -lowQual = 20, -nRate = 0.005, -qualRate = 0.5, other parameters default). Subsequently, the quality-filtered reads were utilized to calculate the K-mer frequency with k = 17, using Jellyfish (v. 2.2.10)^24^ and GCE (https://github.com/fanagislab/GCE). Our estimation resulted in a genome size of 2138.26 Mb, with a peak 17-mer depth of 81. The heterozygosity and repeat rate were found to be 0.33% and 53.52%, respectively (Fig. 1). For the initial genome assembly, we used 30.11 Gb HiFi reads utilizing HiFiasm (v0.16.1)^25^ with default parameters. This preliminary assembly yielded a genome size of 2.30 Gb, with a contig N50 of 123.49 Mb (Table 3).

**Table 3:** Genome assembly information statistics of *E. imbricata*.

Name	scaffold length(bp)	scaffold number	contig length(bp)	contig number
max_len	367,353,949	—	216,744,616	—
N10	367,353,949	1	184,263,635	2
N20	270,538,922	2	145,330,077	3
N30	210,880,045	3	144,276,328	4
N40	165,689,209	4	134,777,127	6
**N50**	**137,212,766**	**5**	**123,485,570**	**8**
N60	126,377,274	7	82,034,986	10
N70	103,468,624	9	44,230,409	14
N80	52,023,501	12	29,028,912	20
N90	24,087,099	19	18,198,761	30
**Total_length**	**2,296,226,205**	**208**	**2,296,181,705**	**297**

**Fig. 2:**
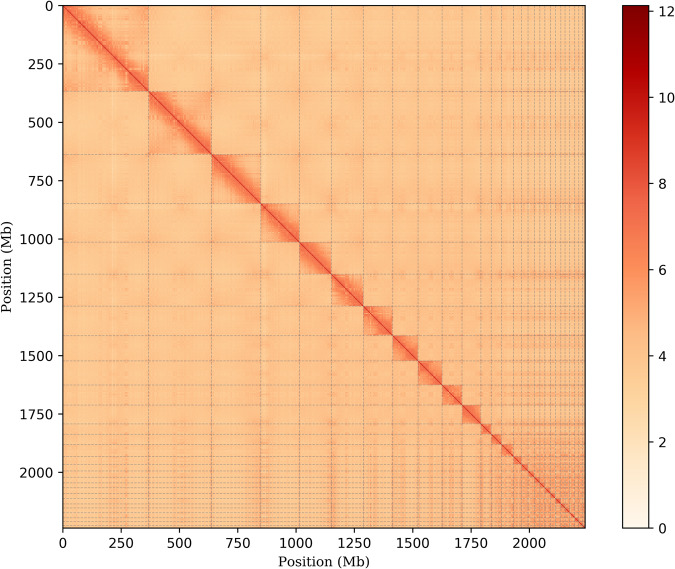
Hi-C interaction heatmap. The genome features of *E. imbricata*: genome-wide Hi-C heatmap of chromatin interaction counts. The color bar indicates contact density from red (high) to white (low).

The paired-end reads obtained from the Hi-C library were mapped to the assembled genome using BWA (v 2.2.1) (parameters: -SP5M) to get the unique mapped paired-end reads, which were used to construct the Hi-C association scaffold^26^. The 3D-DNA pipeline was employed to cluster, sequence, and orient the contigs to construct a genome-wide interaction matrix^27^. Additionally, Juciebox (v1.11.08)^28^ was used for manual error correction, resulting in the final assembly of 28 chromosomes. The quality of the genome assembly was validated by a heatmap of the Hi-C assembly interaction bins, demonstrating excellent results (Fig. 2). The length of the final assembled genome was 2,296,181,705 bp, with a contig N50 of 123,485,570 bp and scaffold N50 of 137,212,766 bp (Table 3). Approximately 2,239,151,156 bp (97.52%) of the assembled result were anchored to 28 pseudochromosomes (Chr) (Table 4).

**Table 4:** The statistics of 28 chromosomes.

Superscaffold	Number of Contigs	Length of Contigs	Length of Superscaffold
**Superscaffold1**	6	367,351,449	367,353,949
**Superscaffold2**	3	270,537,922	270,538,922
**Superscaffold3**	2	210,879,545	210,880,045
**Superscaffold4**	9	165,685,209	165,689,209
**Superscaffold5**	3	136,753,183	136,754,183
**Superscaffold6**	7	137,209,766	137,212,766
**Superscaffold7**	2	126,376,774	126,377,274
**Superscaffold8**	4	108,217,865	108,219,365
**Superscaffold9**	2	103,468,124	103,468,624
**Superscaffold10**	2	85,382,497	85,382,997
**Superscaffold11**	3	80,991,145	80,992,145
**Superscaffold12**	1	44,230,409	44,230,409
**Superscaffold13**	2	43,605,402	43,605,902
**Superscaffold14**	10	52,019,001	52,023,501
**Superscaffold15**	1	33,690,442	33,690,442
**Superscaffold16**	1	28,278,392	28,278,392
**Superscaffold17**	1	26,859,508	26,859,508
**Superscaffold18**	1	24,087,099	24,087,099
**Superscaffold19**	2	21,009,672	21,010,172
**Superscaffold20**	3	24,665,703	24,666,703
**Superscaffold21**	1	20,969,501	20,969,501
**Superscaffold22**	1	23,206,941	23,206,941
**Superscaffold23**	15	17,870,070	17,877,070
**Superscaffold24**	6	20,324,372	20,326,872
**Superscaffold25**	1	21,400,234	21,400,234
**Superscaffold26**	3	17,198,820	17,199,820
**Superscaffold27**	1	16,597,414	16,597,414
**Superscaffold28**	24	10,284,697	10,296,197

### Repeat annotation

To identify tandem repeats and interspersed repeats (transposon elements), we employed a combination of two methods: homology-based and de novo prediction. For the homology-based analysis, RepeatMasker (v4.1.2, -nolow -no_is -norna -parallel 2) and RepeatProteinMask (v1.36, -engine ncbi -noLowSimple -pvalue 0.0001) (http://www.repeatmasker.org) were used to predict TEs within the *E. imbricata* genome based on the known TE protein database and RepBase library (v21.12)^29^. For de novo prediction, we constructed an ab initio repeat sequence library of the *E. imbricata* genome using RepeatModeler (v2.0.2a) and LTR_FINDER (v1.0.5)^30^. RepeatMasker was then used to search and classify the repeat regions against this newly constructed repeat library. Tandem Repeat Finder (TRF)^31^ was utilized to identify tandem repeats, while RepeatMasker was employed to identify non-dispersed repeat sequences. Genome annotation revealed that transposable elements make up approximately 55.51% of the *E. imbricata* genome (Table 5).

**Table 5:** Repeat sequence classification result statistics.

Type	RepeatMasker TEs Length (Bp)	RepeatMasker TEs % in genome	RepeatProteinMask TEs Length (Bp)	RepeatProtein Mask TEs % in genome	De novo Length (Bp)	De novo % in genome	Combined TEs Length (Bp)	Combined TEs % in genome
**DNA**	320993341	13.98	20551028	0.89	56059002	2.44	332199830	14.47
**LINE**	343643983	14.97	215768453	9.4	347924349	15.15	490384998	21.36
**SINE**	36151133	1.57	0	0	21814536	0.95	39867932	1.74
**LTR**	240609432	10.48	38099594	1.66	286540223	12.48	454431615	19.79
**Other**	2020	0	0	0	0	0	2020	0
**Unknown**	16813890	0.73	0	0	35948143	1.57	49400032	2.15
**Total TE**	954077583	41.55	274319132	11.95	717813895	31.26	1274556467	55.51

### Gene prediction

Three strategies were used for *E. imbricata* gene structure annotation: ab initio annotation, homology prediction, and RNA-sequencing-assisted prediction. For homology prediction, we aligned protein sequences from closely related species (*Chelonia mydas*, *Dermochelys coriacea*, *Trachemys scripta elegans*, *Chrysemys picta* and *Gopherus evgoodei*) with *E. imbricata* genome sequence to define gene models using Exonerate (v2.2.0)^32^. Ab initio prediction was generated using Augustus (v3.3)^33^ and Genescan (v1.0)^34^. In addition, RNA-seq data from *E. imbricata* was assembled and aligned to the repeat-masked genome to identify splice sites and exonic regions. All data were then integrated using MAKER (v3.00)^35^. PASA^36^ was used to further refine the gene structure based on transcriptome data. The final comprehensive gene set comprised 20,206 genes (Table 6).

**Table 6:** Statistical analysis of protein coding genes.

Gene set	Number	Average gene length (bp)	Average CDS length (bp)	Average exon per gene	Average exon length (bp)
De novo/AUGUSTUS	19443	34575.77	1606.62	8.9	180.55
De novo/Genscan	29371	52432.85	1429.19	8.51	168.01
homo/*C. mydas*	39707	24526.09	1129.89	5.57	202.68
homo/*D. coriacea*	24984	54511.03	1602.27	8.83	181.4
homo/*T. scripta*	35353	26512.34	1157.39	5.71	202.64
homo/*G. evgoodei*	40252	22972.18	1089.43	5.32	204.64
homo/*C. picta*	38752	23317.54	1083.31	5.43	199.33
trans.orf/RNAseq	8021	22702.63	943.67	6.94	357.99
MAKER	21354	36383.13	1566.33	9.05	221.78
PASA	20206	39185.7	1624.34	9.46	231.05

### Gene function annotation

To perform functional annotation of the integrated gene set, we aligned the genes to several databases, including SwissProt^37^, KEGG^38^, TrEMBL^39^, GO Ontology (GO)^40^, and NR (ftp://ftp.ncbi.nlm.nih.gov/blast/db/FASTA/nr.gz), using Blastp (parameters: -e 1e-5). PfamScan and the InterProScan (v5.35–74.0) were used to search protein structural domains based on the PFAM and InterPro^41^ protein database, respectively. As a result, 99.48% of the predicted protein-coding genes were functionally annotated (Table 7).

**Table 7:** Functional annotation of protein-coding genes for *E. imbricata*.

Type	Number	Percent (%)
Total	20206	
InterPro	17357	85.9
GO	13681	67.71
KEGG_ALL	19888	98.43
KEGG_KO	14515	71.84
Swissprot	19044	94.25
TrEMBL	19924	98.6
NR	20090	99.43
Annotated	20101	99.48

Note: Seven protein databases were used to predict gene functions: Nr, InterPro, Gene Ontology, KOG, KEGG, SwissProt and TrEMBL. The table shows the numbers of genes that were matched to each database.

### Gene family evolution and phylogenetic relationships

**Fig. 3:**
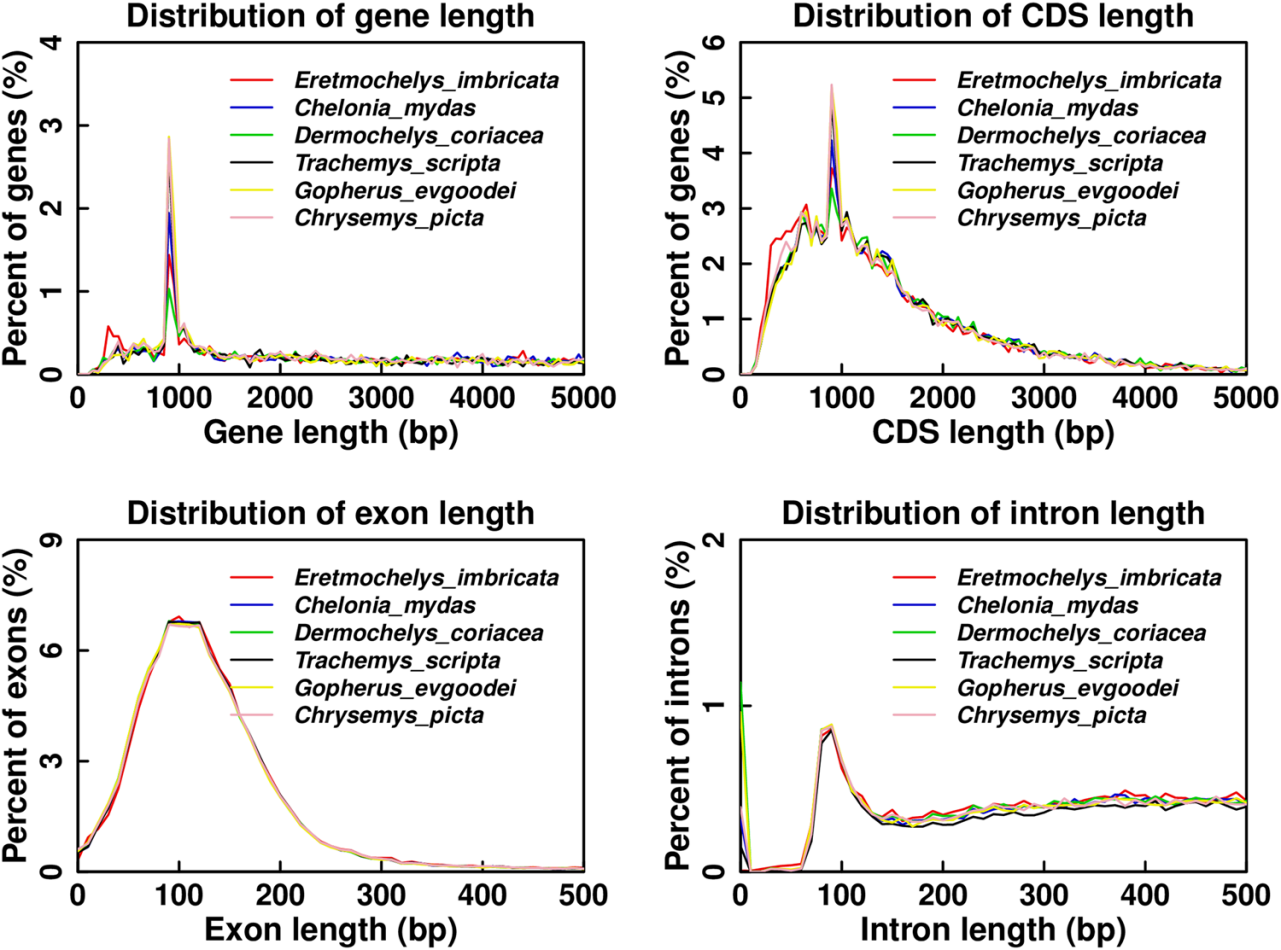
Gene family clustering status classification statistics. Distribution of gene, CDS, exon and intron length for protein-coding genes in *Eretmochelys imbricata* and other turtle genomes.

To identify orthologous gene groups, we conducted a comparative analysis of the protein sequences of *E. imbricata* with those of ten additional species, namely *C. mydas* (NCBI: GCA_015237465.2)^42^, *D. coriacea* (NCBI: GCA_009764565.4), *T. scripta* (NCBI: GCA_013100865.1)^43^, *G. Evgoodei* (NCBI: GCA_007399415.1), *C. picta* (NCBI: GCA_000241765.5), *Gavialis gangeticus* (NCBI: GCA_001723915.1), *Thamnophis elegans* (NCBI: GCA_009769535.1), *Crocodylus porosus* (NCBI: GCA_001723895.1)^44^, *Gallus gallus* (NCBI: GCA_016699485.1)^45^, and *Homo sapiens* (NCBI: GCA_000001405.29). The OrthoFinder2 (v2.5.4)^46^ tool was employed to cluster the genes from the 11 species into gene families using default parameters. After analysis of the gene family, a total of 94.2% (19039) of the 20206 protein-coding genes were clustered into 15,829 orthologous groups in *E. imbricata* (Fig. 3). The average ortholog group contained 1.20 genes per group, and we identified 62 gene families, comprising 320 genes, were found to be unique to *E. imbricata* (Table 8). Additionally, we identified 6,507 single-copy genes based on orthologous genes from the 11 species.

**Table 8:** Species clustering statistics.

Species	Genes number	Family number	Unique families	Single copy	Average genes per family
***E. imbricata***	20206	15829	62	6507	1.203
***C. mydas***	19485	16067	9	6507	1.204
***D. coriacea***	18256	15681	6	6507	1.153
***T. scripta***	17792	14834	8	6507	1.192
***G. evgoodei***	19595	15690	18	6507	1.235
***C. picta***	20319	16113	11	6507	1.243
***G. gangeticus***	13406	12300	3	6507	1.085
***C. porosus***	13978	12776	0	6507	1.088
***T. elegans***	18070	14031	117	6507	1.262
***G. gallus***	17712	13825	108	6507	1.22
***H. sapiens***	19918	14846	300	6507	1.27

**Fig. 4:**
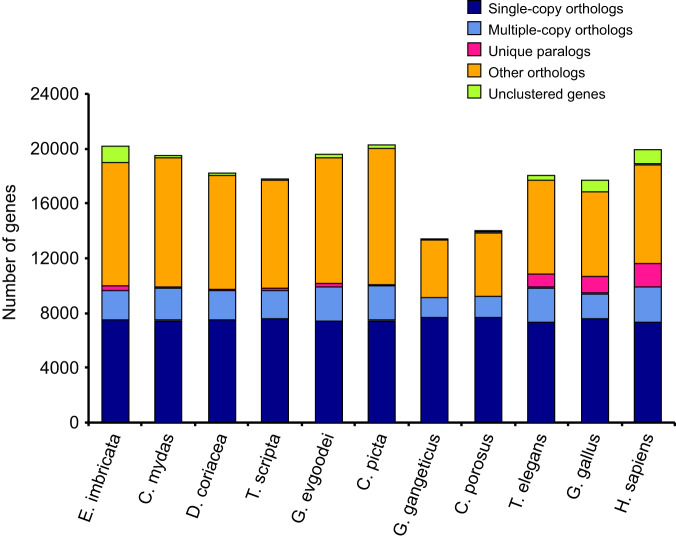
Comparison of orthologous genes between *Eretmochelys imbricata* and 10 other species. Horizontal coordinates represent the species and vertical coordinates represent the number of genes. The dark blue blocks represent single-copy homologues orthologs; the light blue blocks represent multiple-copy orthologs; the red blocks represent unique paralogues; the orange blocks represent other orthologs and the green blocks represent unclustered genes.

To investigate the evolutionary relationships between *E. imbricata* and other sea turtle species, we performed protein sequence alignments for each species’ single-copy orthologues using MUSCLE (v3.8.31)^47^. These alignments were then translated into corresponding coding DNA sequences (CDS). The evolutionary tree was constructed using the maximum likelihood method in RAxML (v8.2.12, parameters: -f a -x 12345 -# 100 -m PROTGAMMAAUTO)^48^. Calibration times were obtained by integrating the constructed evolutionary trees with data from the TimeTree website^49^. Divergence times were estimated using R8s (v1.81, -b)^50^ and the MCMCTree program with default parameters in the PAML (v4.10.0)^51^ packages. The phylogenetic tree reveals the evolutionary relationships between *E. imbricata* and other sea turtle: *D. coriacea* diverged approximately 53.0 million years ago (mya) from a common ancestor with *C. mydas* and *E. imbricata*. In addition, *C. mydas* was the closest sequenced relative to *E. imbricata*, having diverged from their common ancestor around 36.7 to 40.3 mya. (Fig. 4).

### Contraction and expansion of gene families

**Fig. 5:**
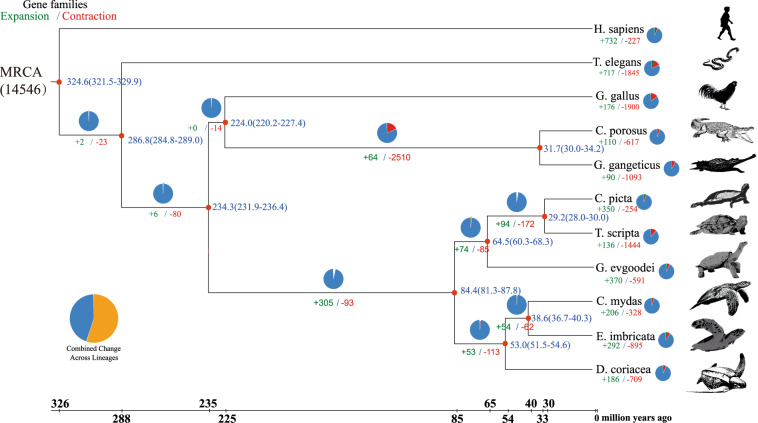
Phylogenetic tree of *E. imbricata* and other species. The maximum likelihood phylogenetic tree based on 6507 concatenated single-copy orthologous genes. The bootstrap value of all nodes is supported at 100/100. Numbers below the branches represent the number of expanded (green) and contracted (red) gene families. The scale at the bottom represents divergence time. The pie chart represents gene families (black, expanded; red, extracted; blue, others).

The time-calibrated phylogenetic tree was utilized to estimate gene family contractions and expansions through CAFÉ (v4.2.1)^52^. In comparison to 10 closely related species, the investigation revealed 292 expanded gene families and 895 contracted gene families in the *E. imbricata* genome (Fig. 5). Further functional annotation of the expanded gene families through GO and KEGG enrichment analyses highlighted their significant involvement in pathways related to olfactory transduction - olfactory receptor, the immune response - pathways for intestinal immune network for IgA production, and detoxification - cytochrome P450.

### Positively selected genes

To gain insights into the selection pressure on the single-copy orthologous genes, the rate ratio (ω) of nonsynonymous (Ka) to synonymous (Ks) nucleotide substitutions was estimated based on the phylogenetic tree using the PAML (v4.10.0)^53^ package. Employing the branch-site model of Codeml^54^ within the PAML package, the rate ratio of the foreground branch of *E. imbricata* and all other branches was determined within the likelihood framework. As a result, a total of 1,487 positively selected genes were identified with a likelihood ratio test (LRT) significance level of ≤0.05 and false discovery rate (FDR) of ≤ 0.05 in the *E. imbricata* genome. The GO enrichment analysis demonstrated significant enrichment in the terms “binding,” “olfactory receptor,” as well as “ECM-receptor” and “Focal adhesion” in the KEGG pathway enrichment analysis.

In summary, we obtained the high-quality chromosome-level genome of *E. imbricata*. The newly generated reference genome will significantly contribute to our understanding of the genetic diversity of sea turtles and facilitate future comparative evolutionary studies and the conservation efforts for this endangered species.

## Data Records

The *E. imbricata* genome project was deposited at NCBI under BioProject No. PRJNA872952. The Illumina sequencing data were deposited under NCBI Accession No. SRR21312391^55^; the PacBio sequencing data were deposited under NCBI Accession No. SRR21311912^56^; the Hi-C sequencing data were deposited under NCBI Accession No. SRR21312300^57^; the RNA-seq data were deposited under NCBI Accession No. SRR21311913^58^; the assembled genome sequence was deposited into NCBI under accession number JARRBA000000000^59^; the genome annotation files are available in Figshare^60^; the phylogenetic and molecular evolution analyses data are available in Figshare^61^.

## Technical Validation

### Genome assembly and gene prediction quality assessment

**Fig. 6:**
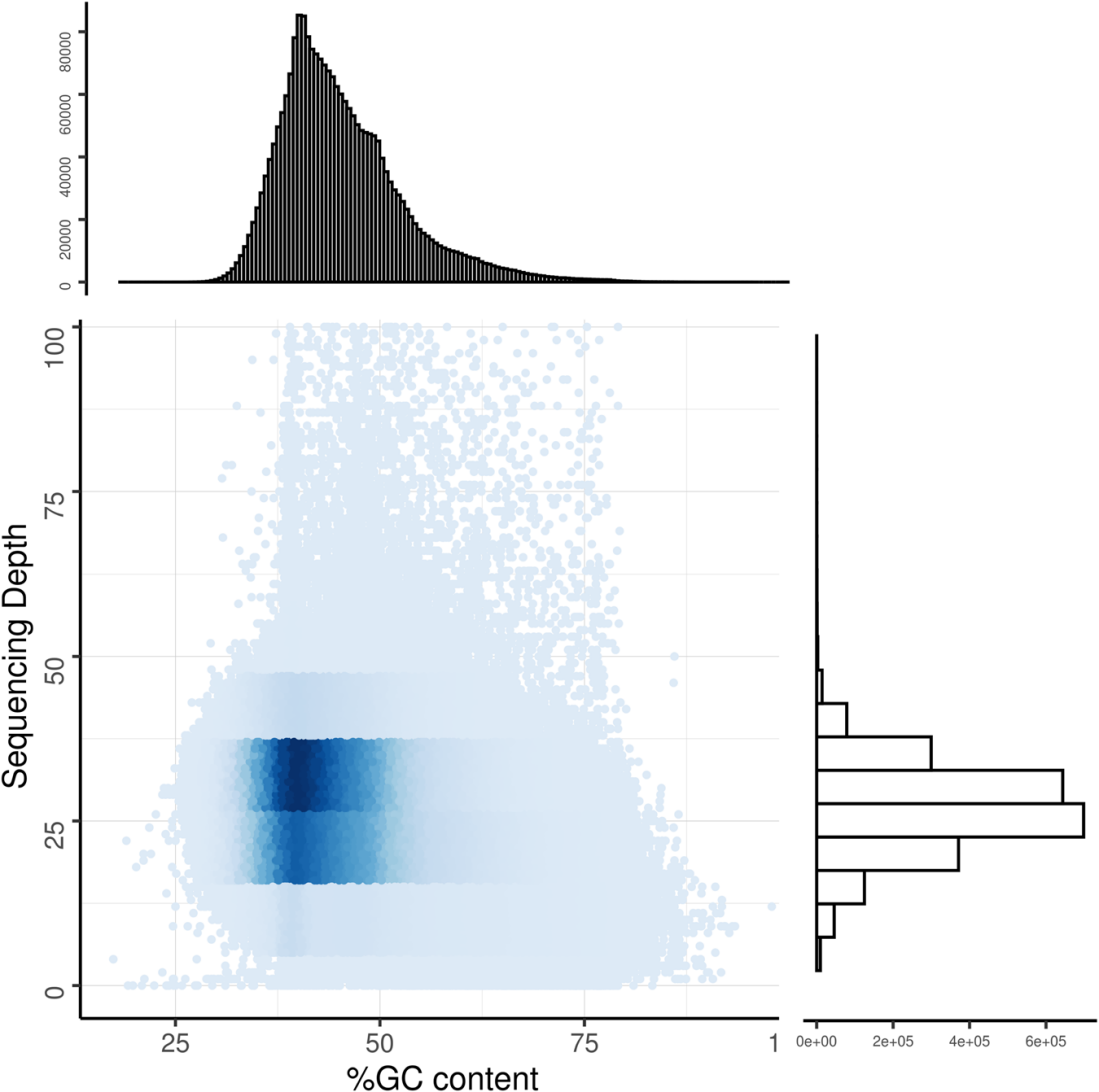
GC content and sequencing depth distribution density map. The x-axis represents the GC content; the y-axis represents the average depth.

The completeness of the *E. imbricata* genome was assessed using BUSCO with the tetrapoda_odb10 (parameters: -m genome -l tetrapoda_odb10)^62^. The assembled genome exhibited approximately 97.4% complete BUSCO genes, with 96.8% being complete and single copy, 0.6% being complete and duplicated, 0.7% being fragmented, and 1.9% being missed (Table 9). Minimap2 (v2.12, parameters: -ax map-pb)^63^ aligned the assembly results with HiFi data to obtain the depth of coverage for each locus on the genome, which showed mapping and coverage rate were estimated to be 100% and 99.85%, respectively (Table 10). Subsequently, employing 1000 bp non-overlapping sliding windows along the chromosomes, we calculated the GC content and the average depth of reads (Fig. 6). Collectively, all of the above results indicate that we have obtained a high-quality genome of *E. imbricata*.

**Table 9:** Genome completeness assessment of *E. imbricata* using BUSCO.

BUSCO	Number	Percent (%)
Complete	5,173	97.4
Complete single copy	5,141	96.8
Complete duplicated	32	0.6
Fragmented	35	0.7
Missing	102	1.9
Total	5,310	100

**Table 10:** Statistics of HiFi and Hi-C data mapped to genome.

Data type	Mapping rate (%)	Average sequencing depth	Coverage (%)	Coverage (> = 5X,%)	Coverage (> = 10X,%)	Coverage (> = 20X,%)
**BGI**	99.51	82.6	99.55	99.23	98.93	98.23
**PacBio**	100	27.26	99.98	99.85	99.09	88.66

## Usage Notes

All data analyses were performed according to the manual and protocols of the published bioinformatic tools. The version and parameters of software have been described in Methods section.

## Data Availability

No specific code or script was used in this work. Commands used for data processing were all executed according to the manuals and protocols of the corresponding software.
